# Interactive CO-learning for Research Engagement and Education (I-COREE) Curriculum to Build Capacity Between Community Partners and Academic Researchers

**DOI:** 10.31372/20180304.1030

**Published:** 2018

**Authors:** Connie Kim Yen Nguyen-Truong, Roschelle L. Fritz, Junghee Lee, Christine Lau, Cang Le, Jane Kim, Holden Leung, Thai Hien Nguyen, Jacqueline Leung, Tuong Vy Le, Anthony M. Truong, Julie Postma, Renee Hoeksel, Catherine Van Son

**Affiliations:** a College of Nursing in Vancouver, Washington State University, Vancouver, WA, USA; b School of Social Work, Portland State University, Portland, OR, USA; c Asian Health & Service Center, Portland, OR, USA; d Asian/Pacific Community; e College of Public Health and Human Sciences in Global Health, Oregon State University, Corvallis, OR, USA; f College of Nursing, Washington State University, Spokane, WA, USA

**Keywords:** community, academic-community partnership, building capacity, trust, rapport, culturally safe, sustainability, education, training, curriculum

## Abstract

The voice of diverse communities continues to be minimal in academic research. Few models exist for education and training of new research topics and terminology and building partnership capacity in community-engaged research. Little is known about integrative education and training when building participatory research partnerships for sustainability and developing trust and rapport. Community partners at an Asian community-based health and social services center in a large metropolitan area wanted to explore the cultural context of a health-assistive smart home that monitors and auto-alerts with changes in health. With historical and recent rising trends in culturally insensitive research in several diverse communities, the concept of technology-enabled monitoring in the privacy of one’s home brings uncertainty. Academic nurse researchers and community partners co-created a culturally safe integrative education and training curriculum, the Interactive CO-learning for Research Engagement and Education (I-COREE). The purpose was to design, implement, and evaluate the curriculum to respond to the community partners’ needs to create a culturally safe space through an integrative education and training to facilitate building partnership capacity for research engagement including developing trust and rapport and addressing uncertainties in health-assistive technologies. Popular education tenets informed the curriculum. Twelve academic and community partners participated, four were team teachers who co-led the session. Implementation of the experiential, multimodal co-learning activities were conducted within ahalf-day. The curriculum evaluation indicated that it helped bridge critical conversations about partners’ fears of the unknown, approach culturally sensitive topics safely, and trust and rapport. Key elements may be translatable to other partnerships.

The voice of culturally diverse communities continues to be minimal in academic research. Community-engaged culturally responsive researchers must respond to community partners’ needs and create a culturally safe space to facilitate building partnership capacity for research engagement including the development of trust and rapport (i.e., relationship) so that mutually beneficial partnerships can be developed, and research opportunities established. A long-term relationship is needed in an academic-community research partnership.

A culturally safe space for education and training of new research topics and terminology is needed between academic researchers and community partners. Few models exist that help with this, or with building partnership capacity for sustainability in community-engaged research (Belone et al., 2016; Calderón, Norris, Hardigan, Calderón, & Hays, 2015; Tan et al., 2014). Furthermore, little is known about integrative education and training (using cognitive, affective, and psychomotor domains) when building participatory research partnerships for sustainability and developing trust and rapport (Farquhar, Michael, & Wiggins, 2005; Nguyen, Tang, & Hsiao, 2017; Wiggins et al., 2009). Various communication challenges can interfere with building sustainable academic-community partnerships. Differences in communication styles stemming from personal or professional opinions and varying levels of education can influence the quality of communication (King et al., 2015). Additionally, a lack of understanding about the roles of experts, academic versus community resources, and organizational concerns may lead to poor conflict resolution and dissolution of an academic-community partnership (Calderón et al., 2015).

Thus, academic nurse researchers at a large public university in the Pacific Northwest in the United States of America (U.S.A.) and culturally diverse community partners at an Asian community-based health and social services center in a large metropolitan area co-created a culturally safe integrative education and training curriculum, the *Interactive CO-learning for Research Engagement and Education* (I-COREE). The I-COREE curriculum is an educational innovation project. The purpose of this project was to design, implement, and evaluate the I-COREE curriculum to respond to community partners’ needs to create a culturally safe space. The integrative education and training curriculum facilitated building academic-community partnership capacity for research engagement that included developing trust and rap-port and addressing uncertainties in health-assistive technologies.

The I-COREE curriculum met two of the community partners’ needs for a culturally safe space: the need to (1) build partnership capacity for research engagement and (2) co-learn in a shared cultural context that utilized health-assistive smart home monitoring (i.e., Smart Home) research language. Community partners wanted to explore the potential influence of culture on perceptions of the Smart Home (^1^Nguyen-Truong & ^1^Fritz, 2018). The Smart Home uses sensors to detect residents’ movement within the home and artificial intelligence (i.e., computer algorithms capable of performing tasks that typically require human intelligence) to identify residents’ everyday activities (Cook, Crandall, Thomas, & Krishnan, 2012) so that health assessments can be auto-generated (Dermody & Fritz, 2018; Fritz & Dermody, 2018; Sprint, Cook, Fritz, & Schmitter-Edgecombe, 2016). Community partners wanted to know more about the Smart Home because most minority older adults remain at home or are cared for by family members (American Association of Retired Persons, 2014) and because community members may benefit from Smart Home technology. With historical and recent rising trends with culturally insensitive research methodologies and implementations in several diverse communities (Bozzo, Bali, Evaniew, & Ghert, 2017; Centers for Disease Control and Prevention, 2015; Li et al., 2018; Skloot & Turpin, 2010), the perception of researchers placing motion sensors in one’s home brings uncertainty. Including minority data in emerging artificial intelligence algorithms is an urgent need. In this article, we describe the pedagogical approach to design, implementation, and evaluation on the use of the I-COREE curriculum as an education and training method (i.e., perspectives and experiences of community partners and academic researchers). Limitations and future recommendations for curriculum use are discussed.

## Approach

### Design

#### 

##### Pedagogical approach to the I-COREE curriculum

Tenets of popular education (also known as empowerment education) informed the main concepts of the I-COREE curriculum, including holistically addressing cognitive, affective, and psychomotor domains. Popular education tenets included in the curriculum are: co-learning with our heads (cognitive), hearts (affective), and bodies (psychomotor); creating a learning environment of trust and assisting community partners to openly share their perspectives and experiences; promoting equality between community partners and academic researchers; validating knowledge gained through life experience is equally important as knowledge gained through formal education; actively participating in the learning process; and using artistic teaching modalities as important tools for teaching (Wiggins, 2012). Reflection as a group on interactive, multimodal co-learning activities throughout the implementation of the I-COREE curriculum is central. Evaluating the co-learning experience is also central. After implementing the I-COREE curriculum, partners evaluate strengths, utility, and areas for improvement.

### Implementation

#### 

##### Setting and partners as co-learners

This educational innovation project was determined to be ex-empt by a local university Institutional Review Board. The I-COREE curriculum was conducted with community partners at the Asian community-based health and social services center within a half-day (3.5 h) session. A total of twelve partners participated, of which two were academic researchers and ten were community partners including the Chief Operating Officer (senior community administrator), Program Managers (other senior community administrators), and Community Health Education Specialists who met in a large meeting room conducive to seeing partners when seated and moving around comfortably. The Chief Operating Officer selected the ten people who attended. All identified as Asian (Chinese, Vietnamese, and Korean), and among them, eleven were also first generation immigrants. Of the twelve partners who attended, four were partner-team teachers who were bilingual and bicultural. They collectively led the implementation of the I-COREE curriculum as well as participated alongside other community partners (i.e., team teachers were an academic nurse scientist who was Vietnamese, a social worker academic researcher who was Korean, the Chief Operating Officer who was Chinese, and the Program Manager who was Vietnamese). A Vietnamese bicultural and bilingual clinical nurse from the Asian community had the role as a trained designated observer (details on evaluation procedures are described later). The I-COREE curriculum was conducted in English.

##### Methodological underpinnings of the I-COREE curriculum

The I-COREE curriculum implementation consists of four modules (details on the step-by-step processes of the experiential, multimodal co-learning activities are described in Table 1). To begin, the academic nurse researcher invites partners to share thoughts and cultural insights about the activities that includes *dinámicas*, which are social learning activities to facilitate developing trust and rapport (Wiggins, 2012), group discussions, role playing, and mock scenarios. This ongoing group reflection is threaded throughout the I-COREE curriculum implementation phase. The intent is to encourage open and authentic conversation between community partners and academic researchers for trust and rapport building. Partners are encouraged to embrace one’s own culture and be astute to power relations and that of others. The I-COREE curriculum Module I focuses on *Celebrating Cultural Capital* and *Shared Cultural Context and Research Language* (American Nurses Association, 2015; Fritz, Corbett, Vandermause, & Cook, 2016; Israel, Eng, Schulz, & Parker, 2012; Nursing Council of New Zealand, 2011; Rogers, 2003, 2004). Taking this time to celebrate partners’ culturally-specific and health-assistive technology expertise and resources from both community and academic partners as a collective (i.e., cultural capital) honors partners who are *at the table* for their unique skills, talents, commitment, and future contributions in the Asian immigrant community and the science of gerontechnology. Partners can keep this in mind in the introduction of new topics including difficult terminology and concepts (e.g., what is a Smart Home and its features and functions) during the review of the study protocol via simulated co-learning activities (i.e., role playing) regarding study procedures that contains those terms and concepts. The I-COREE Curriculum Module II focuses on the *Team Approach to Translation* and *Multi-Cultural and Multi-Lingual Expertise* (Cook, Schmitter-Edgecombe, & Dawadi, 2015; Fritz et al., 2016; Israel et al., 2012; Nguyen-Truong, Nguyen et al., 2017; Nguyen-Truong, Pedhiwala et al., 2017; Nursing Council of New Zealand, 2011). Achievement of meaningful cross-cultural translation and interpretation of terms and concepts including language that does not exist in the targeted Asian languages using a team approach is essential for rigor so that misunderstandings and miscommunication does not occur. Layering health-assistive technology features and functions add complexity to cross-cultural translation and interpretation in respective languages and building a sense of connectedness through shared experiences promote de-isolation. The I-COREE Curriculum Module III underscores the *Empowered Asian Immigrant Minority Voice Through a Participatory Approach to Data Collection and Analysis*, *Scientific Group Level Assessment Method*, and *Shared Decision-Making* (Lincoln & Guba, 1985; Nguyen-Truong, Nguyen et al., 2017; Rae & Green, 2016; Vaughn & Lohmueller, 2014). Envisioning what it might be like to engage older Asian immigrants as study participants through use of mock scenarios (i.e., step-by-step data collection and analysis procedures) regarding participation in group discussion and analysis of their own perceptions with the group facilitator in real time encourages anticipation of challenges and discussion on how to promote study participants’ empowerment in using their voice. This may help to promote open dialogue and sharing of partners’ experiences and strategies. The I-COREE Curriculum Module IV is integrated throughout the other modules and regards *Science and Community Outreach as a Journey and Celebrating Leadership Moments in the Community-Engaged Research Process*. Involving academic and community partners’ in dissemination is important in outreaching and meaningful sharing about their work as a partnership so that others may learn. This also includes formally recognizing individuals and as well as a collective regarding contributions. Ongoing conversations about what kind of information is shared and why, to who, and what warrants co-authorship or being acknowledged for community and scholarly dissemination is needed so that communication is open, genuine, and transparent.

In Table 1, we describe key elements of the I-COREE curriculum implementation, including: (a) learning objectives, (b) education and training topics organized as four modules, (c) the step-by-step processes (i.e., methods) of the experiential multimodal co-learning activities (i.e., interactive), and (d) the Plus/Delta evaluation questions that capture co-learners’ perspectives of their experience as part of a group discussion that occurs following the implementation phase. Each module contains references that informed the multimodal co-learning activities (i.e., read, handson, visuals).

**Table 1:** Learning Objectives, Topics, Multimodal Co-Learning Activities, and the Step-by-Step Processes of the Interactive CO-Learning for Research Engagement and Education (I-COREE) Curriculum

Learning objectives	Topics in the modules	Multimodal co-learning activities and the step-by-step processes
**I-COREE Curriculum Module 1** (1) Encourage partners to interact while learning about one another.	**Connectedness Through** **Storytelling**	**Favorite Season Storytelling Dinámica (Social Learning Activity)** Storytelling about a favorite season to encourage connection through conversation for developing trust and rapport. A partner (team teacher in the education and training) may start first to model the dinámica activity. Pass around an item (e.g., squishy ball) to indicate whose turn is next for storytelling. “Hi, my name is ___________ and pronouns are ___________.” “I am from [affiliation] in [city, state].” “My favorite season is ___________.” “[Describe why].” Invite partners to share their thoughts about the dinámica activity.
(2) Review the study protocol and honor partners *at the table* for their formal and lived experience expertise and establish a shared understanding.	**Celebrating Cultural Capital:** **Research Team and Study** **Overview**	**Shared Cultural Context in Research Language** Provide handouts on study protocol and materials. Review and discuss research team’s study timeline, roles and meaningful involvement in various research activities throughout a community-engaged, participatory, and a values-based research approach. Use of a conceptual framework, in alignment with the community and academic agreement, study purpose, methods, and dissemination; include tenets of community-engaged research, tenets of Roger’s Diffusion of Innovation theory for adoption in an identified community, and values-based nursing principles of autonomy, the right to self-determination, and providing culturally safe care for individuals and minority populations. (American Nurses Association, 2015; Fritz, Corbett, Vandermause, & Cook, 2016; Israel, Eng, Schulz, & Parker, 2012; Nursing Council of New Zealand, 2011; Rogers, 2003; Rogers, 2004)
(3) Simulate co-learning through practice and observation.	**Study Procedures Part 1: Outreach, Recruitment, and Informed Consent Process**	**Role Playing: Informed Consent Process—Eligibility Screening Script and Study** **Information Form** Have a community partner as the interviewer and a partner-team teacher as the potential study participant. Other partners as observers. This encourages a supportive, culturally safe, and co-learning environment.
(4) Facilitate discussion and anticipation of scenarios with the informed consent process.	**Study Procedures Part 1: Group Share**	**Debriefing on the Role Playing: Informed Consent Process—Eligibility Screening Script and Study Information Form** Invite partners to discuss research definitions and procedures, which includes voluntary participation and confidentiality and de-identification; share cultural insights.
(5) Stimulate co-learning through practice and observation.	**Study Procedures Part 2: Online Sociodemographic Questionnaire**	**Role Playing: Interviewing Study Participants—Online Sociodemographic Questionnaire** Have a community partner as the interviewer and a partner-team teacher as the potential study participant. Other partners as observers. This encourages a supportive, culturally safe, and co-learning environment.
(6) Encourage discussion and anticipation of scenarios using a web-based format to enter responses.	**Study Procedures Part 2: Group Share**	**Debriefing on the Role Playing: Interviewing Study Participants—Online Sociodemographic Questionnaire** Invite partners to discuss research definitions and procedures, which includes consistency and confidentiality and de-identification; share cultural insights.
**I-COREE Curriculum Module II**		
(1) Encourage partners to co-learn through open discussion of similar and diverse cultural and language perspectives.	**Utilization of a Multi-Cultural Lens for Cross-Cultural Translation and Interpretation of the -Research Language that is non–existent in Targeted Asian Languages**	**“I See This and That – We Hear You – Thank You” Dinámica (Social Learning Activity)** Provide pictures related to the research topic illustrating terms and concepts and engage in group discussion to encourage trust and rapport building. Invite partners to break out into groups according to cultural and language expertise and each group take a picture (i.e., hand held visual) to view, and then show to the other partners. A partner-team teacher may start first to model the dinámica activity. “[Speak in a language other than English] I see a [item].” “[Speak in a language other than English] [Describe item in photo].” “[Interpret in English] I see a [item].” “[Interpret in English] [Describe item in photo].” Respond as a group, “We hear you. Thank you.” Invite partners to share their thoughts about the dinámica activity. (Fritz, Corbett, Vandermause, & Cook, 2016; Cook, Schmitter-Edgecombe, & Dawadi, 2015.)
(2) Support partners’ interactions through honoring diverse perspectives and resolving ambiguities through open discussion to achieve consensus.	**Study Procedures Part 3: Team Approach to Translation**	**Multi-Cultural and Multi-Lingual Expertise** Community partners and academic research partners with cultural and language expertise and experience in English and targeted Asian languages. Partners share power in leadership in the translation and interpretation of the study protocol and materials as a team. Partners are encouraged to understand one’s own culture and power relations and that of others for a culturally safe environment. (Israel, Eng, Schulz, & Parker, 2012; Nguyen-Truong, Nguyen et al., 2017; Nguyen-Truong, Pedhiwala et al., 2017; Nursing Council of New Zealand, 2011).
(3) Encourage discussion and anticipation of scenarios using a team approach to translation.	**Study Procedures Part 3: Group Share**	Invite partners to discuss research definitions and procedures, which includes a team process for meaningful, credible, trustworthiness, in translation; share cultural insights.
**I-COREE Curriculum Module III**		
(1) Encourage partners to interact while sharing travel memoirs.	**Ongoing Relationship and Trust Building Through Storytelling and Movement**	**Traveling Through Time Memoirs Storytelling and Movement Dinámica (Social Learning Activity)** Storytelling about a memorable moment in one’s travel, for example in the State of residence, United States, or to one’s home country or another country and continent, or a travel wish accompanied with a movement related to that travel. This encourages trust and rapport building through conversation. A partner-team teacher may start first to model the dinámica activity. Pass around an item (e.g., picture frame) to indicate whose turn is next for storytelling. “My memorable moment was when I travelled to ___________.” “[Describe why].” Or “My wish is to travel to ___________.” “[Describe why].” “One related movement from that travel is ___________.” [encourage partners to join in to do the same travel related movement]. Invite partners to share their thoughts about the dinámica activity.
(2) Review the scientific qualitative group level assessment methodology with partners for a shared understanding of the method, which is more participant driven.	**Empowered Asian Immigrant Minority Voice Through a Participatory Approach to Data Collection and Analysis with Study Participants in Real Time: An Overview**	**Scientific Group Level Assessment (GLA) Method: Shared Decision-Making** The participatory group discussion method includes study participants’ responses in real time and interactively analyzing the data with study participants and research team to co-construct meaning. The overview encourages discussion among partners about consistency in the following six steps to ensure rigor: (1) climate setting to build trust and rapport, (2) visual presentation to study participants on health-assistive smart homes—before the step on generating data, (3) appreciating perspective, (4) reflecting, (5) understanding the data, and (6) selecting themes. (Lincoln & Guba, 1985; Nguyen-Truong, Nguyen et al., 2017; Rae & Green, 2016; Vaughn & Lohmueller, 2014)
(3) Invite partners to anticipate how it might be for study participants (older Asian immigrants) to participate in a qualitative investigation: responding to study participants’ questions, for partners to accurately document the responses, and how to interactively analyze the data with study participants in real time and as a research team and as an iterative and reflexive manner.	**Study Procedures Part 4: Participatory Approach to Data Collection and Analysis**	**Walk-Through Mock Scenarios: Participatory Approach to Data Collection and Analysis** Partners are encouraged to think about the fluidity in the interaction and participatory group discussion method. A partner-team teacher describes the procedure and refers to the Semi-Structured, Open-Ended Interview Question Guide and use of separate pieces of flipchart paper to document study participants’ responses. *Climate Setting Step*. Building trust and rapport through an activity prior to data collection and analysis with study participants. *Generating Data Step*. A partner-team teacher reads a prompt aloud and discusses with partners on ways to engage study participants to share their thoughts. A discussion on the use of probing and following-up questions to help expand on responses. This process is repeated for prompts. For example, (1) “Desires/Concerns” related to…; (2) “I need to consider this before I move forward with having a…”; and (3) “In order to be effective at employing a _______ in the Asian immigrant community, then you should…”.
		*Appreciating Perspective Step.* A partner-team teacher discusses with partners on ways to engage study participants such as those who may not have participated and for ideas on how to facilitate the addition of thoughts after they have moved through the prompts. *Reflection Step. A partner-team teacher discusses with *partners on providing time for *study* participants to take time to think to themselves about what the data as a whole means to them. *Understanding the Data Step.* A partner-team teacher discusses with partners the process of reviewing responses to each prompt on the flipcharts. Study participants will be asked to: (a) look for common themes from the content (data); (b) analyze from their perspective; and (c) interactively and collaboratively analyze data about major themes through discussion with the group facilitator. *Selecting Themes Step.* The partner-team teacher discusses with partners the process of collaboration with *study* participants on selecting and prioritizing themes. After this process, the partner-team teacher describes how the research team will engage in additional analysis and interpretation of the data about shared and unique understandings of the data. (Lincoln & Guba, 1985; Rae & Green, 2016; Vaughn & Lohmueller, 2014)
(4) Foster discussion and anticipation of scenarios using a participatory group discussion including interactively analyzing the data with study participants and research team.	**Study Procedures Part 4: Group Share**	Invite partners to discuss research definitions and procedures, which includes a participatory process for credible, trustworthiness, in data collection and analysis, potential bias; share cultural insights.
**I-COREE Curriculum Module IV is Integrated Throughout Modules I, II, and III**		
(1) Encourage partners to interact while recognizing leadership moments in rigorous team research.	**Science and Community Outreach as a Journey**	**Celebrating Leadership Moments in the Community-Engaged Research Process** Invite partners to share their role and leadership moment or to describe the role and leadership moment of other partners in the community-engaged research process. “My role as a ___________ and leadership moment in ___________.” Or “[name a partner] role as a ___________ and leadership moment in ___________.”
(2) Engage partners as co-authors/presenting co-authors through early and ongoing thoughtful conversations that includes anticipation and actual substantial, meaningful contributions.	**Navigating the Dissemination Journey**	**Integrating Early and Ongoing Conversations that are Anticipatory and Actual Substantial, Meaningful Contributions** Preparation of manuscripts for refereed, academic journals. Preparation of an educational presentation on findings to faculty and students at a large public university; Asian community leaders and members, and community-at-large; communicating research conference, and grant funders.
(3) Facilitate discussion and anticipation of substantial, meaningful involvement in conceptualization of ideas, implementation, analysis and interpretation, AND writing, and co-authorship.	**Navigating the Dissemination Journey: Group Share**	Invite partners to discuss research definitions and procedures, which includes engagement of partners in all types of dissemination; share cultural insights.
**Group Evaluation on I-COREE Curriculum**		
**Plus/Delta Evaluation: Open-Ended, Semi-Structured Questions**		How did the interactive co-learning activities stimulate co-learning? How useful were the co-learning activities? What can be improved to help our co-learning? Other thoughts that you would like to share? (O’Connell & Vandas, 2015, pp. 34–35)

### Educational Project Evaluation

The academic nurse researcher conducted the Plus/Delta evaluation (adapted from O’Connell & Vandas, 2015) following the completion of all four I-COREE curriculum modules. This evaluation occurred as a group discussion where community partners as co-learners shared their perspectives and experiences focusing on the strengths (i.e., pluses that helped with co-learning), utility (i.e., usefulness of the co-learning activities), and recommendations (i.e., deltas to help with co-learning) of the I-COREE curriculum. An open-ended, semi-structured question guide (see Table 1, Group Evaluation) was used to facilitate this discussion. The clinical nurse, trained in the art of observation, documented group dynamics and partners’ questions as well as their evaluation responses. The academic nurse researcher met the clinical nurse afterwards and reviewed field notes that were kept during the implementation phase. Then the academic nurse researcher verified content and summarized comments.

## Outcomes

### Emotional and Psychological Safe Communication Space for Introducing New Topics

Community partners indicated that the I-COREE curriculum helped to bridge critical conversations between community partners and academic researchers about community partners’ fears of the unknown as they engaged in the interactive, multimodal co-learning activities. Community partners had not previously been exposed to the Smart Home as a health-assistive technology. The academic researchers shared their experiences to illustrate the potential challenges with research in this area and how these challenges might be mitigated when working with the Asian immigrant community. Community partners’ concerns included, (1) not knowing what this technology is and its capabilities, (2) language related to the new technology does not exist in the targeted languages (e.g., Chinese, Vietnamese, and Korean), (3) lack of clarity regarding ways to engage in outreach and recruitment of older Asian immigrants who are unaware of this technology, and (4) how to implement the research alongside academic researchers that lead to meaningful outcomes. Community partners indicated that they needed to feel emotionally and psychologically safe and comfortable in engaging and implementing the research with older Asian immigrants alongside academic researchers. Academic partners wanted community partners to feel safe, and to not experience feeling invalidated, apprehensive, or misunderstood. The researchers wanted to decrease the chance for miscommunication. Academic researchers demonstrated sensitivity when they reviewed the study protocol and materials while working intentionally with community partners to create a safe communication space. Academic researchers started with acknowledging and commending what community partners already knew based on previous extensive work with older Asian immigrants in the community. They also acknowledged the trusting relationship that had already been built with older Asian immigrants. Partners discussed academic-community partnership values, which include a long-term relationship, commitment in health equity by having the voice of older Asian immigrants, and meaningful contribution to the Asian immigrant community and the science of gerontechnology. Through participating in the interactive, multimodal co-learning activities that contained terms and concepts of the new research topic throughout, community partners reported feeling more at ease with the terminology, in their interactions with academic researchers, and their readiness to commence the research.

### Approaching Culturally Sensitive Topics Safely

Partners discussed that older Asian immigrants are highly respected and regarded as elders in the community. Partners were sensitive to the historical trauma, which included awareness that community partners’ worked with several older Asian immigrants who had been forced to leave their homelands due to war and had experienced physical and mental torture (e.g., difficult living situation, starvation, fear of death) as captives of war and being *monitored* during imprisonment. For example, community partners worked with older Vietnamese immigrants who previously held high profile positions in Vietnam (e.g., professors, journalists) and experienced being a prisoner due to expressing different personal values. Here, the I-COREE curriculum helped to bridge critical conversations between community partners and academic researchers. Community partners raised a concern on how to engage older Asian immigrants as study participants, specifically if they did not speak up about being *monitored* for health reasons (a culturally sensitive topic) during the group discussion. Partners envisioned what it would be like for older Asian immigrants to participate by role-playing and using the mock scenario co-learning activities in the I-COREE curriculum. The activities helped to inform an in-depth discussion where community partners reported on the following priorities with regard to engaging older Asian immigrants while considering their need to feel emotionally and psychologically safe, and to engage in a comfortable dialogue. Priorities included: allowing for time to process before responding, using inclusive questions to elicit meaningful data, *“sit in silence”*, providing listening support (older Asian immigrant participants may need to speak their thoughts prior to actually responding), anticipating and allowing further questions or comments about the unknown research topic following the group discussion, and minimizing interruption of the group discussion by limiting English interpretation—*“Need to let it [group discussion] be natural”*.

### Trust and Rapport

Community partners shared that they found the I-COREE curriculum to be useful in developing trust and rapport with academic researchers. Specifically, they appreciated co-learning activities of storytelling, the cross-cultural translation and interpretation of terms and concepts of health-assistive technology that do not exist in the targeted Asian languages, using hand held visuals, and cultural expectation in research participation.

Community partners shared that storytelling enhanced engagement, connectedness, and relationship building with academic researchers, specifically the *favorite season* and *traveling through time memoirs dinámicas*. Partners talked about how much they learned about and from each other through these *dinámicas* (i.e., social learning activities) and that this was a part of the enjoyable and meaningful education and training experience. One community partner indicated, *“We know each other. We can speak in the group and do movements as a group.”* Another community partner described, *“We use dinámicas in our trainings with community health workers. We are willing to do it.”* Community partners felt comfortable sharing their experiences working with Asian immigrant community subgroups throughout the I-COREE curriculum modules, as they observed academic researchers being genuinely interested in working with older Asian immigrants.

Community partners shared that they felt empowered when translating and interpreting difficult non-existing terms and concepts of health-assistive technology into the targeted languages. In particular, they felt empowered with the *I See This and That – We Hear You – Thank You dinámica* that involved use of various hand-held visuals. Academic researchers discussed use of a rigorous cross-cultural team approach for meaningful translation and interpretation. Community partners felt their efforts were validated in using a team approach within their respective cultural and language expertise. Community partners shared that they tend to focus on their own language expertise, and this helped them to feel connected to others. While standing in a circle in smaller groups by self-identified culture and language expertise, community partners held the visuals and provided interpretations of the item, and then discussed their interpretation decisions. The item was then translated from the targeted language into English. Community partners reported that they liked the stated group response, *“we hear you - thank you,”* when they were encouraged to speak in both languages because the emphasis was placed on collaboration. A collaborative approach meant staying focused and listening actively to avoid misunder-standings and miscommunication about the terms and concepts. One community partner said, *“Learn to know each other. Interpret[ing] can be challenging.”* Another said, *“Encouraging, getting approval from the group.”* Still another community partner noted that this approach, *“Encourages speaking our language.”* Partners were able to observe the supportive interactions within and across the cultural and language groups. One community partner described, *“Although we speak different languages, our body language is important. When they speak in different language, I focus on body language…I finally understand when they speak the common language [English], then I focus on meaning.”*

Community partners shared their cultural expectations regarding research participation. They shared their previous cultural experiences engaging with older Asian immigrants to promote a welcoming atmosphere for building trust and rapport. They emphasized the importance of honoring differences across Asian ethnic subgroups in research. For example, a Vietnamese subgroup might enjoy a minimal dance activity for building trust and rapport prior to the group discussion, while another ethnic group may prefer a completely different activity. A community partner said, *“It depends on the group. For example [Korean], research equals serious.”* Partners discussed letting senior community administrators, program managers, or specialist staff from each group discussion provide meaningful climate setting to build trust and rapport, and to intentionally welcome older Asian immigrants (i.e., Vietnamese group—a minimal dance exercise, Korean group—an arm stretching activity, and Chinese Cantonese and Chinese Mandarin groups—engage in light general talk and laughter).

## Limitations

While there is a structured process for the experiential multimodal co-learning activities of the I-COREE curriculum, critical to successful implementation is the ability for academic researchers to create a culturally safe space for communication with and among community partners. In this way, team teachers who are unfamiliar with the research topic can engage by *learning by teaching* alongside the academic nurse researcher. A limitation is that this innovative educational project is new and has only been used one time with one community organization. Additionally, it was implemented in one half-day session, which accommodated schedule needs, but more time was needed for group reflection and discussion than anticipated (i.e., additional 30 min). Another limitation is that the co-learning activities focused on the targeted languages of Chinese, Vietnamese, and Korean. These languages reflected the older Asian immigrant groups primarily served by community partners at the Asian-based community health and social services center but language and cultural expectations in research may be different for other groups. This educational innovation project was carried out in the Pacific Northwest of the U.S.A. in a large metropolitan area. Community identified needs may be different for other minority groups (i.e., other racial-ethnic groups) and for communities with varying geographic locations (e.g., urban versus rural).

### Future Recommendations for Curriculum Use

Future users of the I-COREE curriculum should establish understandings regarding how community partners would like to learn and work collaboratively with academic partners. Additionally, they should assess and address unique community partners’ needs (i.e., associated with minority-specific historical traumas, topic concerns, and more) to create an intentional safe communication space. Key elements of the I-COREE curriculum identified here may be translatable to other academic-community partnerships. We recommend this curriculum be tailored and used in other academic-community partnerships to bridge critical conversations. Community or academic partners using the I-COREE curriculum in the future should consider the following: duration and number of the integrative education and training sessions (be responsive to community partners’ needs); diverse learning styles through use of interactive, multimodal co-learning activities; academic researchers and the community partners co-lead and team teach; and conducting education and training in an environment at the partners’ location that is conducive for seeing partners when seated and moving around comfortably.

### Conclusion

The views and needs of culturally diverse populations need to be understood if research is to meet the community’s needs. To conduct research alongside culturally and linguistically diverse populations, academic researchers must establish a relationship of trust and rapport with community partners that supports a safe space for communication. In this article, we described the design, implementation, and evaluation of community partners’ experiences using a culturally safe integrative educational and training curriculum (I-COREE) to build the foundation for long-term research collaboration related to health assistive technologies. Academic researchers and community partner team teachers intentionally worked alongside each other as equals while co-leading the implementation of the I-COREE curriculum. The curriculum consisted of learning objectives, four modules, and step-by-step processes for a variety of multimodal co-learning activities. Partners shared thoughts and cultural insights about the activities that included *dinámicas* to facilitate developing trust and rapport, as well as group discussions, role playing, and mock scenarios. A foundation of trust and rapport between academic and community partners was built through ongoing group reflection of the interactive nature of the co-learning activities. The I-COREE curriculum helped to bridge relationships, which is foundational to a sustainable partnership and meaningful research. Curriculums such as I-COREE can be instrumental in developing partnerships with culturally diverse communities so that they can be sustainable over time.

## Acknowledgments

The authors are grateful to the Program Managers and Community Health Education Specialists at Asian Health & Service Center for their engagement in the project. The authors are also appreciative of the anonymous peer reviewers for assistance.

## Declaration of Conflicting Interests

The authors declared no potential conflicts of interest with respect to the research, authorship, and/or publication of this article.

## Funding

Dr. Connie Kim Yen Nguyen-Truong, PhD, RN, Alumnus PCCN, and Dr. Roschelle L. Fritz, PhD, RN were awarded the Beta Psi Chapter of Sigma Theta Tau International – Naomi Ballard Nursing Research Award and Washington State University Vancouver Research Mini-Grant that supported the work.
